# Original and Mirror Face Images and Minimum Squared Error Classification for Visible Light Face Recognition

**DOI:** 10.1155/2015/842084

**Published:** 2015-10-21

**Authors:** Rong Wang

**Affiliations:** Changchun University of Science and Technology, Changchun 130022, China

## Abstract

In real-world applications, the image of faces varies with illumination, facial expression, and poses. It seems that more training samples are able to reveal possible images of the faces. Though minimum squared error classification (MSEC) is a widely used method, its applications on face recognition usually suffer from the problem of a limited number of training samples. In this paper, we improve MSEC by using the mirror faces as virtual training samples. We obtained the mirror faces generated from original training samples and put these two kinds of samples into a new set. The face recognition experiments show that our method does obtain high accuracy performance in classification.

## 1. Introduction

The conventional minimum squared error (MSE) algorithm has been widely used for pattern recognition and this algorithm also performs well in face classification. MSEC [1, 2], respectively, takes the sample and its class label as the input and output and tries to obtain the mapping that can best transform the input into corresponding outputs. MSE has many advantages in classification. This method is simple and easy to operate. MSEC not only is suitable for two-class classification [3], but also can be applied to multiclass classification [4–6]. MSEC has been extended to nonlinear methods. Kernel MSE (KMSE) [7, 8] is a well-known nonlinear extension of MSEC. Ruiz and Lopez-de-Teruel [9] proposed a KMSE method in which the solution is based on generalized inverse of the kernel matrix. Differing from MSE, KMSE tries to obtain nonlinear mapping between the input and output. A “Lasso” method based on MSE (LMSE) [10–12] has been proposed for pattern recognition. LMSE tries to obtain a good performance by minimizing the *l*_1_ norm of the solution vector and can be viewed as an extension of conventional MSEC. The total least squares (TLS) is also a well-known improvement to the MSE. TLS [13, 14] assumes that both the input and output are corrupted and each of them can be expressed as sum of the corresponding “true data” and “measurement noise.” Other people also proposed another method [15–19] to improve this MSE algorithm. For example, Xu et al. [15] proposed a modified minimum squared error (MMSE) to improve MSE. Besides pattern recognition, MSE has been applied for other fields such as clustering, data fitting, and density estimation [20–23]. Moreover, the well-known representation based methods can be viewed as generalized MSEC methods, for example, collaborative representation classification (CRC) [24], two-phase test samples sparse representation (TPTSSR) [25], and sparse representation-based classifier (SRC) [26].

The MSE algorithm first uses training sample and its class label of learning mapping and exploits the obtained mapping to predict the class label of testing sample [27, 28]. Then, MSE chooses the training sample that is the nearest to the test sample. Finally, MSE assigns the testing sample into class that this training sample belongs to.

The conventional MSEC is limited by the number of training samples. And the images in face recognition were faced with some problems, such as variations of illumination, facial expression, and poses. Facial expression and poses variations can be dealing with the restrict condition to reduce errors, but illumination variation is hard to control [29–31]. Therefore, dealing with illumination problem is necessary for face recognition. Shang et al. proposed an illumination face recognition algorithm based on ordinal feature [32], adopted ordinal feature as the ordinary variable. The solutions of illumination invariable face recognition [33–36] can be classified into three kinds, method based on the normal features, modeling based on changed illumination, and having a standard condition for illumination.

It seems that more training samples are able to reveal more possible variations of the illumination, facial expression, and poses and beneficial for correct classification of the face. However, in real world, it is hard to capture enough samples and a system usually has a limited number of training samples. In order to obtain better face classification, previous literatures have proposed synthesizing new samples from the true face image. These new samples were called virtual samples [37, 38]. For example, Tang et al. [39] proposed prototype faces and an optic flow and expression ratio image based method to generate virtual samples. Ryu and Oh [30] exploited the distribution of the given training set to generate virtual samples. Jung et al. [40] synthesized new face samples with virtual training samples. Xu et al. [41] used the average face of every two original training samples to generate virtual training samples. Liu et al. [42] exploited kernel principal component analysis (PCA) and symmetrical faces to classify the test samples. Xu et al. [43] used symmetrical faces as training samples for two-step face recognition. There are many advantages for generating virtual images. We can obtain features from virtual faces that the original samples do not have, though there are many similar features between virtual faces and original training samples. Besides this, adding new training sample can compensate for the limited training samples.

In this paper, our method proposed the MSE classification based on mirror faces [44, 45] for face recognition. We first establish the equation of the MSE and solve it. Then, we exploit the obtained solution to predict the class labels of test samples. Finally we classify the test sample and obtain the accuracy of classification. In this paper, we use three databases to do experiments, PIE database, Yale B database, and subset of Yale database. Our method has a higher classification accuracy than conventional MSE. This paper will describe the following parts.  Section 2 introduces our proposed method, Section 3 analyzes our method, Section 4 shows the results of experiments, and Section 5 is the conclusion.

## 2. The Proposed Method

In this section we will present the main steps of our proposed method in detail. Suppose that there are *c* classes and each class has *m* training samples. There are *n* numbers of total training samples (*n* = *c∗m*). Let *x*_1_,…, *x*_*n*_ represent all the training samples and we assign a class label to each class.

### 2.1. Main Steps of the Proposed Method

The proposed method includes five steps. The first step generates mirror faces of original training samples. The second step puts both the mirror faces and original training samples into a new set and obtains the class label of each new training sample. The third step uses the new training samples to perform MSE algorithm and obtain mapping. The fourth step predicts the class label of testing sample for face recognition. The last step gets the ultimate classification result. And we present these steps as follows.

**Step 1 .**

Obtain mirror face of each training sample. Let *x*_*i*_ be *i*th training sample in the form of training image matrix; *x*_*i*_ is *p* × *l* matrix. And *x*_*i*_ = (*y*_1_, *y*_2_, *y*_3_,…, *y*_*l*_), where *i* ∈ (1,2,…, *n*), and *y*_*j*_  (*j* = 1,2,…, *l*) is the column vector of *x*_*i*_. Set variable *z*, where $z_{j}={\left[\begin{array}{cccc} y_{1\;l-j} & y_{2\;l-j} & \cdots & y_{p\;l-j} \end{array}\right]}^{T}$. The mirror face of *x*_*i*_ is defined by $\bar{x_{i}}=(z_{1},z_{2},\ldots ,z_{l})$.

**Step 2 .**

Use both original training samples and mirror faces to structure a new training set and sign its class label matrix. The new training sample number *N* = 2*n*. Transforming every original training sample *x*_*i*_ and the mirror face $\bar{x_{i}}$ into *p* × *l*-dimensional column vector, we can obtain (*p* × *l*) × *N*-dimensional training samples matrix $X=(x_{1},\bar{x_{1}},x_{2},\bar{x_{2}},\ldots ,x_{n},\bar{x_{n}})$. We use *c*-dimensional vector *b*_*i*_, *i* = 1,…, *N*, to represent the class label of each training sample and the class label matrix *b* defined as $b^{T}=\left(\begin{array}{cccc} b_{1} & b_{2} & \cdots & b_{N} \end{array}\right)$; for the definition of *b*_*i*_ please see Section 2.2.

**Step 3 .**

Use the new training sample set to perform MSE algorithm face recognition. From this step, we will obtain mapping for test samples. For the algorithm, please see Section 2.2.

**Step 4 .**

Exploit the mapping matrix obtained in Step 3 to classify the test samples, and predict the class label of test sample which the training samples are nearest to.

**Step 5 .**

Exploit the result obtained by conventional MSE and get the classification result. Combine the original class label of test sample with its predicted class label in step three. If the same, then we consider that the test sample is correctly classified. Finally, we can obtain the accuracy of face recognition.

### 2.2. Minimum Squared Error (MSE) Algorithm

In this subsection, we present the algorithm of MSE face recognition. There, we use *c*-dimensional vector *b* to represent the class label for each class. If a sample is from *j*th class, then *j*th element of its class label is one and the other elements are all zeros. For example, if the sample is from the first class, we will take *b*_1_ = [1 0 0 ⋯ 0] as its class label.

There, we assume that matrix *W* can transform each training sample into its class label; MSE has the following equation:

(1) $$\begin{array}{c} XW=b, \end{array}$$

where

(2) X=x1⋮xN,b=b1⋮bN.

We refer to *W* as transform matrix, and *b*_*i*_ is the class label of the *i*th training sample. As (1) cannot be directly solved, we convert it into the following equation:

(3) $$\begin{array}{c} X^{T}XW=X^{T}b. \end{array}$$

We can obtain *W* using

(4) $$\begin{array}{c} \bar{W}={\left(\;X^{T}X+\gamma I\;\right)}^{-1}X^{T}b, \end{array}$$

where *γ* and *I* denote a small positive constant and the identity matrix. MSE classification classified test sample *y* in the form of row vector as follows: the class label of *y* is first predicted using the following equation:

(5) $$\begin{array}{c} b_{y}=y\bar{W}. \end{array}$$

Then, the distance between *b*_*y*_ and the class labels of the *c* classes is calculated. We choose the minimum distance which means *b*_*y*_ is the closest to the class label of the *k*th class, and then *y* will be classified into *k*th class.

## 3. Analysis of the Proposed Method

**Figure 1:**
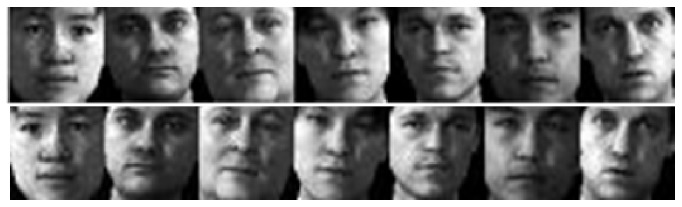
Some original training samples from the PIE database and the mirror faces generated from the original training samples. The first row shows the original training samples. The second row shows the mirror faces generated from the original training samples.

**Figure 2:**
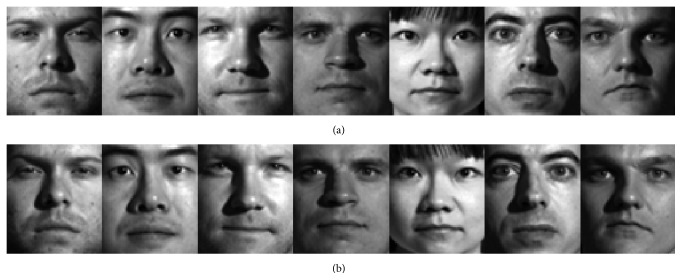
Some original training samples from the Yale B database and the mirror faces generated from the original training samples. The first row shows the original training samples. The second row shows the mirror faces generated from the original training samples.

**Figure 3:**
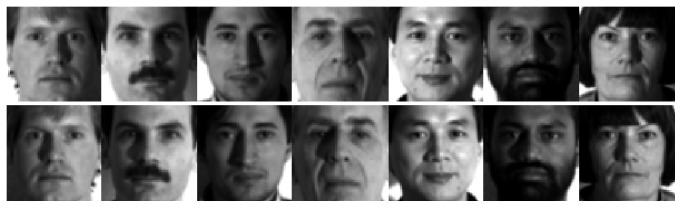
Some original training samples from the Yale database and the mirror faces generated from the original training samples. The first row shows the original training samples. The second row shows the mirror faces generated from the original training samples.

**Figure 4:**
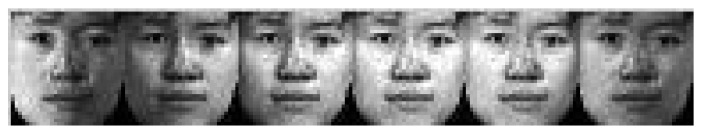
Some test samples from the PIE face database from the same class.

**Figure 5:**
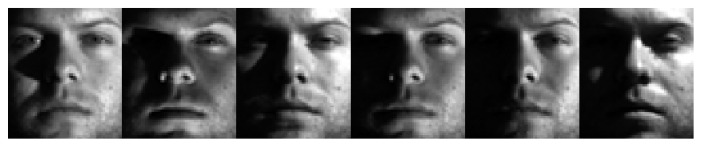
Some test samples from the Yale B face database from the same class.

**Figure 6:**
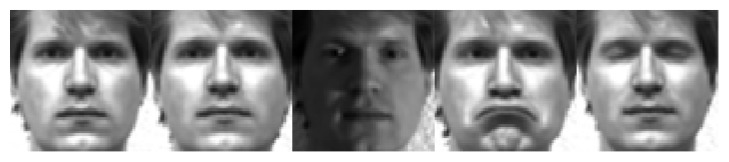
Some test samples from the Yale face database from the same class.

**Figure 7:**
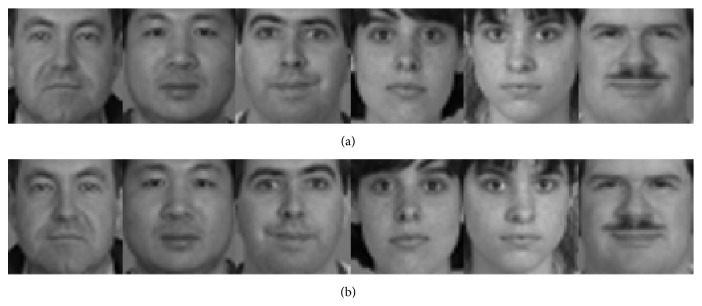
Some original training samples from the FERET face database in the same illumination condition and the mirror faces generated from the original training samples. The first row shows the original training samples. The second row shows the mirror faces generated from the original training samples.

In this section we show the rationales of the proposed method. First, the mirror faces in the proposed method indeed reflect some possible appearance of the face, which are not shown by the original training samples.  Figure 1 shows some original training samples from the PIE face database and the mirror faces generated from the original training samples.  Figure 2 shows some original training samples from the Yale B face database and the mirror faces generated from the original training samples.  Figure 3 shows some original training samples from the Yale database and the mirror face images generated from the original samples. It seems that mirror training samples have different illumination and features in comparison with the original training samples. Our method uses both original training samples and their mirror training samples. And from the results of experiments, the mirror face training samples are really beneficial for correct classification of the test sample.  Figure 4 shows some test samples from the PIE face database from the same class.  Figure 5 shows some test samples from the Yale B face database from the same class.  Figure 6 shows some test samples from the Yale face database from the same class. Mirror faces generated from the original samples do not show high accuracy classification in all case; this is only playing well on different illumination.  Figure 7 shows some original training samples from the FERET face database in the same illumination condition and the mirror faces generated from the original training samples. We can see that mirror face training samples have not shown other obvious features. PIE database and Yale B database are all better for our experiments.

The other rationale of the proposed method is that it uses MSE algorithm for face recognition. MSE is easy to calculate and reduce the effect caused by negative errors. Our method uses all training samples and tries to minimize the sum of the deviation between the obtained class labels and true class labels. MSEC is able to convert every training sample. By mapping (4), we obtained predicted class label of test sample, where *γ* = 0.01. Then, we choose the minimum distance class label as its class label. Finally, we judge the accuracy of classification. As a result, by using mirror face training samples we can increase the probability of test sample being correctly classified.

## 4. Experimental Results

In this paper, we use three databases to conduct experiments. The first database is a subset of the PIE [46] database whose samples are only influenced by illumination. The second database is Yale B database [47]. And the third database is Yale database [48].

### 4.1. Experiments on PIE Database

From the PIE database, we used 68 subjects and each subject has 21 gray images. Every image was resized to a 32 × 32 image. For the PIE database, we adopted three cases to do experiments. In the first case, we used the first image of each subject as original training samples and took the remaining images as the test samples. In the second case, we used the first two images of each subject as original training samples and took the remaining images as the test samples. In the third case, we used the first three images of each subject as training samples.  Table 1 shows the experimental results on PIE database. From the results, we can see that our method shows greatly accuracy on classification compared to conventional MSEC. With increasing quantity of training samples, the accuracy of classification has improved. When the original training samples are up to three, the classification rate almost reached full percent.

### 4.2. Experiments on Yale B Database

The Yale B database includes 38 subjects and each subject has 64 gray images. Every image was resized to a 96 × 84 image. We used five cases of original training samples to do experiments; we took the first 3, 4, 5, 30, and 35 face images of each subject as the original training samples and treated the remaining images as the test samples.  Table 2 shows the results of Yale B database experiments. It shows again that our proposed method achieved higher accuracy classification than conventional MSEC.

### 4.3. Experiments on Yale Database

We used a subset of the Yale database to test our method. This subset consists of 165 images from 15 subjects; each subject includes 11 images. Every image was resized to 32 × 32. We, respectively, took first 2, 3, and 4 face images of each subject as the original training samples and took the remaining face images as the test samples.  Table 3 shows the experiments on the Yale database. According to the experimental results, our method shows better than conventional MSEC, especially for conventional MSEC which only uses mirror images as training samples.

## 5. Conclusions

We propose a very promising method to exploit limited training samples for MSEC face recognition. The new training samples generated in this paper can well exploit the mirror structure of the face. The mirror face is helpful for overcoming the drawback of limited training samples in the real-world face recognition system. And the MSEC is able to obtain high classification accuracy as the training sample nearest to the test sample can provide useful information for classifying it.

## Figures and Tables

**Table 1:** Rate of classification errors (%) on the PIE database.

Number of the original training samples per class	1	2	3
Conventional MSEC using original images	14.78	7.59	0.98
Conventional MSEC using mirror images	83.09	84.21	79.58
Our method	1.32	0.23	0.16

**Table 2:** Rate of classification errors (%) on the Yale B database.

Number of the original training samples per class	3	4	5	30	35
Conventional MSEC using original images	54.92	50.09	48.66	31.27	31.22
Conventional MSEC using mirror images	75.63	74.65	73.95	48.53	41.02
Our method	46.64	39.08	36.08	20.90	16.79

**Table 3:** Rate of classification errors (%) on the Yale database.

Number of the original training samples per class	2	3	4
Conventional MSEC using original images	39.26	34.17	23.81
Conventional MSEC using mirror images	68.15	60.00	42.86
Our method	34.81	29.17	20.95
